# Modelling the Survival of Acid-Adapted and Nonadapted *Escherichia coli* O157:H7 in *Burkina*: A Western African Traditional Fermented Milk Product

**DOI:** 10.1155/2023/1011319

**Published:** 2023-11-20

**Authors:** Leticia Donkor, Nicole Sharon Affrifah, Angela Parry-Hanson Kunadu, Samuel Kwofie, Stephen Yeboah, Bernard Kuditchar

**Affiliations:** ^1^Department of Food Process Engineering, School of Engineering Sciences, College of Basic and Applied Sciences, University of Ghana, P. O. Box LG 77, Legon, Accra, Ghana; ^2^Department of Nutrition and Food Science, College of Basic and Applied Sciences, University of Ghana, P. O. Box LG 134, Legon, Accra, Ghana; ^3^Department of Biomedical Engineering, School of Engineering Sciences, College of Basic and Applied Sciences, University of Ghana, PMB, Legon, Accra, Ghana; ^4^Department of Computer Engineering, School of Engineering Sciences, College of Basic and Applied Sciences, University of Ghana, P. O. Box LG 1181, Legon, Accra, Ghana

## Abstract

*Burkina*, a traditional fermented dairy product, is consumed in most parts of West Africa, including Ghana. Studies on the microflora of *Burkina* have indicated the presence of *Escherichia coli* and other pathogenic organisms. Thus, predicting the survival of *E. coli* in the product will inform the best manufacturing and handling practices. This study investigated the combined effect of storage temperature and time on the survival of acid-adapted and acid-non-adapted *E. coli* O157:H7 in *Burkina*. Samples were pasteurised and inoculated with acid-adapted or acid-non-adapted *E. coli* O157:H7. They were stored at 5, 15, and 30°C for 0, 2, 4, 6, 8, 10, 12, 14, 18, and 48 h, and the bacteria colonies were enumerated. Growth rate (survival versus time) models were developed using MATLAB software. Observed data were fitted to the Baranyi model using the DMFit curve fitting software. The *E. coli* O157:H7 strain appeared inherently tolerant to acid, with storage time having the most significant effect on the response parameter, survival (log CFU/mL). A negative correlation was observed for the primary models (survival versus time), which accounted for 79-97% of the relationship (*p* < 0.05). Although *E. coli* survived, its growth was inhibited over time regardless of acid adaptation.

## 1. Introduction

The growth potential of microorganisms in food has recently been evaluated and predicted by applying mathematical models that serve as promising tools for risk assessment [1, 2]. Such predictive microbiology strategies help describe microorganisms' responses to environmental conditions such as pH and temperature.

*Burkina* is a nutritious fermented milk product composed of steam-cooked agglomerates of millet and spontaneously fermented milk, also known as *nunu* in Ghana. It is similar in consistency and consumption to yoghurt [3].

Although widely consumed, the product has no standardised processing protocols with controlled hygienic practices and critical control points. This limits the potential target market for the product since it does not appeal to many potential consumers who avoid the product due to the perception of poor handling practices during processing and sales [4]. In addition, various studies have raised concerns about the safety of *Burkina* [5–7]. These studies have indicated the presence of bacteria such as *Enterobacter*, *Klebsiella*, *Escherichia coli*, *Proteus vulgaris*, *Staphylococcus*, and *Shigella* in *Burkina*. Although not all foodborne microorganisms are harmful, *Escherichia coli* O157:H7 in food can cause medium to gastrointestinal illness in humans when consumed. Studies carried out between 2000 and 2006 by the Foodborne Diseases Active Surveillance Network of the United States of America reported 3464 *E. coli* O157:H7 infections, among which 218 persons developed haemolytic uremic syndrome and 33 people had died [8]. Another study detected high levels of the bacterium in the food and water of a South African region [9].

The World Health Organisation (WHO) provided global estimates of the burden of foodborne diseases for thirty-one (31) food hazards, which cause two diseases [10]. Six hundred million foodborne illnesses and 420,000 deaths recorded in 2010 were caused by these 31 hazards. Eighteen (18) million disability-adjusted life years (DALYs) of these diseases were attributed to foodborne diarrheal disease-causing agents, including enterohaemorrhagic *Escherichia coli* (EHEC) [10]. For this reason, assessing the growth potential of pathogenic microorganisms in food is essential to reduce the risk of foodborne diseases when consumed. This necessitates models that describe changes associated with microbial growth along the food-handling chain [11].

With the increasing consumer demand for *Burkina*, there is a need to predict storage temperature and time conditions to prevent contamination after processing. Considering this, the study developed mathematical models that predict the survival of *Escherichia coli* O157:H7 in *Burkina* as a function of temperature and time.

## 2. Materials and Methods

### 2.1. Chemicals and Cultures

Unless specified, all reagents used in this study were ordered from an Oxoid supplier (UK). Pure strains of *E. coli* O157:H7 were obtained from the Microbiology Research Laboratory of the Department of Nutrition and Food Science, University of Ghana. The bacterium was previously isolated from *wagashi* (a traditional unripe cheese consumed in Ghana). The bacteria culture was activated twice on nutrient agar, then sterile Tryptic Soy Broth (TSB) at 37°C for 24 h.

### 2.2. Acid Adaptation

Acid adaptation was carried out according to the method described by Ryu et al. [12]. Briefly, 1% glucose w/v was added to TSB before sterilisation at 121°C for 15min. The addition of glucose was to aid acid formation and accumulation following fermentation by *E. coli*, resulting in a drop in pH. The same procedure was followed except for glucose addition for the nonadaptation process. Approximately 1 mL of 24-hour culture broth was transferred into TSB and Tryptic Soy Broth +1% Glucose (TSBG) for nonadaptation and acid adaptation, respectively. The cultures were incubated at 37°C for 18 h to reach the exponential phase. To confirm acid adaptation, 1 mL of acid-adapted and non-acid-adapted cells was separately cultured in 9 mL of sterile TSB acidified with lactic acid to pH 4.0 and incubated at 30°C for 24 h. Cell viability was tested on prepoured MacConkey agar plates at 0, 2, 4, 6, and 24 h of incubation.

### 2.3. *Burkina* Sample Preparation and Inoculation

*Burkina* was obtained from a local University of Ghana Legon campus vendor. The samples were transported to the laboratory on ice at 4°C. The samples were homogenised using a high-speed blender for 5min. Aliquots (100 mL) of the samples were dispensed into sterile screw-cup containers and pasteurised at 63°C for 30min using a water bath set at 70°C, then cooled.

An aliquot (1 mL) of either acid-adapted or non-acid-adapted culture was added to the pasteurised *Burkina* to achieve a final concentration of 10.49 and 10.42 log CFU/mL, respectively.

*Burkina* samples inoculated with acid-adapted (AA) and non-acid-adapted (NA) *E. coli* O157:H7 were thoroughly mixed and stored at 5, 15, and 30°C for 48 h. Storage temperatures were selected to mimic refrigeration and likely temperature abuse during street hawking and consumption.

### 2.4. Microbiological Analysis

At each selected sampling time (0, 2, 4, 6, 8, 10, 12, 14, 24, and 48 h), 1 mL of each inoculated sample was added to 9 mL of 0.1% sterile peptone water in a test tube and vortexed for 2 min using a vortex mixer.

The homogenates (1 mL) were serially diluted in 9 mL of 0.1% sterile peptone water. After that, 100 *μ*L of the suspensions was spread on MacConkey agar and incubated at 37°C for 24-48 h before enumeration.

The pH was measured for samples at the selected temperatures and sampling times (0, 2, 4, 6, 8, 10, 12, 14, 24, and 48 h) using the Mettler Toledo pH meter.

### 2.5. Modelling

One hundred twenty data points were collected at the selected storage temperatures (5, 15, and 30°C) for the duplicated experiments. Primary models were developed using linear regression in MATLAB software 2018 using Equations (1) and (2) as shown below:

(1) $$\begin{array}{c} y=\alpha -\beta x, \end{array}$$

(2) $$\begin{array}{c} y=\alpha +\beta_{1}x+\beta_{2}x^{2}, \end{array}$$

where *y* is the survival (log CFU/mL), *x* is the incubation time, and *α*, *β*_1_, and *β*_2_ are the estimated coefficients derived from the model.

The Baranyi model was used to fit the observed data points using the DMFit Excel add-in (version 3.5) to obtain the maximum growth rate from

(3) $$\begin{array}{c} \ln\left(N\left(t\right)\right)=\ln\left(N_{o}\right)+\mu_{\max}A\left(t\right)-\ln\left[1-\frac{e^{\mu_{\max}A\left(t\right)}-1}{e^{\left(N_{\max}-N_{o}\right)}}\right], \end{array}$$

where ln(*N*(*t*)) is the log of cell concentration at time *t* (h) (CFU/mL), ln(*N*_*o*_) is the log of initial cell concentration (CFU/mL), *μ*_max_ is the exponential growth rate (log/CFU/mL/h), and ln(*N*_max_) is the log of maximum cell concentration.

Root mean squared error (RMSE) values were used to estimate the mean difference between the observed and predicted data points to measure the bias factor. RSME was calculated using

(4) $$\begin{array}{c} \text{RSME}=\sqrt{\frac{\sum {\left(\mu -\mu^{*}\right)}^{2}}{n}}, \end{array}$$

where *n* is the number of observations, *μ* is the empirical values, and *μ*^∗^ is the predicted values.

### 2.6. Statistical Analysis

All means and standard deviations were tabulated for observation using Microsoft Excel. Variance analysis (ANOVA) was conducted using the Minitab software version 17. Tukey's pairwise comparison and Duncan's multiple range tests were used to analyse the significance between means at *α* = 0.05.

## 3. Results and Discussion

### 3.1. Acid Adaptation

The average pH of *Burkina* samples before acid adaptation was 6.71 ± 0.01 and 6.98 ± 0.00 for TSBG and TSB, respectively (Table 1). After the adaptation process, pH values for the 18-hour culture were reduced to 4.75 ± 0.01 (TSBG) and 6.14 ± 0.01 (TSB) (Table 1). *E. coli* fermented the added glucose, resulting in acid accumulation and lowered pH. The results were like earlier studies by Deng et al. [13], who recorded a pH of 4.8 ± 0.2 and 6.2 ± 0.1 for TSBG and TSB cultures after 18-hour fermentation. Ryu et al. [12] also reported 4.9 ± 0.2 and 6.2 ± 0.2 for TSBG and TSB 18-hour cultures, respectively.

### 3.2. Tolerance and Survival of *E. coli*

Overall, *E. coli* O157:H7 concentration decreased with increasing storage time at each storage temperature, irrespective of acid adaptation. Initial cell cultures inoculated into the *Burkina* samples were 10.42 ± 0.05 and 10.49 ± 0.08 log CFU/mL for TSB and TSBG, respectively (Table 2), with detection of the organism in *Burkina* after 48 h signifying the survivability of the organism in the product. Previous studies have also reported decreasing counts of *E. coli* in foods with time [13–15], supporting the observations made.

Before the pasteurisation process, the estimated concentration of *E. coli* was 4.40 ± 0.51 log CFU/mL; however, no growth was detected after pasteurisation. Tsai and Ingham [16] stated that the dose levels required to cause infection of *E. coli* O157:H7 are very low and should not be present in foods. Hence, although decreasing in counts, the concentrations detected after 48 h were ≤2 logs, which can render the product unsafe if the initial population of *E. coli* O157:H7 in the food is high.

Acid adaptation of *E. coli* O157:H7 has been said to augment its survivability in acidic foods. Tosun et al. [17] reported the enhanced survival of acid-adapted *E. coli* O157:H7 in symbiotic and strained-type yoghurts compared to nonadapted controls. On the other hand, similar growth patterns were observed between the nonadapted and adapted *E. coli* O157:H7 in the *Burkina* samples. The difference in cell concentration was not statistically significant (*p* = 0.086). Deng et al. [13] also reported similar growth patterns for acid-adapted and nonadapted *E. coli* O157:H7 in reconstituted infant rice cereal. This similar growth pattern observed for the nonadapted *E. coli* O157:H7 to the adapted could have been because the strain had already adapted to the acidic environment of the fermented dairy product from which it was isolated, proving its tolerance to acid. Moreover, the strain could have been inherently resistant to low acids and may not have required acid adaption for survival. Foster [18] stated that some microorganisms that can grow under neutral pH have specific controlled pH homeostasis systems that make them survive when they are transferred into acidic environments, and *E. coli* is one of the organisms with the best systems. *E. coli* has well-developed glutamate and arginine decarboxylases whose metabolites enhance acid tolerance [18, 19], and this must have contributed to the tolerance of nonadapted *E. coli* in the sample. Various studies have reported the tolerance and survivability of *E. coli* O157:H7 in yoghurt. Guruya et al. [20] reported that nonadapted *E. coli* O157:H7 (NAEC) in traditional yoghurt survived for up to 35 days, and Hudson et al. [14] also reported the survivability of *E. coli* in plain yoghurt for up to 17 days. Although enumeration of the bacteria in *Burkina* did not continue beyond 48 h, the high concentrations observed after 48 h of storage at 5 and 15°C, as presented in Table 2, suggest tolerance of acid-adapted and nonadapted *E. coli* inoculated into the *Burkina* samples.

### 3.3. Storage Temperature and Survival of *E. coli*

AAEC and NAEC in samples stored at 5°C and 15°C survived better than those stored at 30°C (Table 2). There was no statistical difference between survival at storage temperatures of 5°C and 15°C (*p* = 0.948). However, there was a statistical difference between storage temperatures of 15°C and 30°C (*p* = 0.01) and 5°C and 30°C (*p* = 0.01), irrespective of acid adaptation.

**Figure 1:**
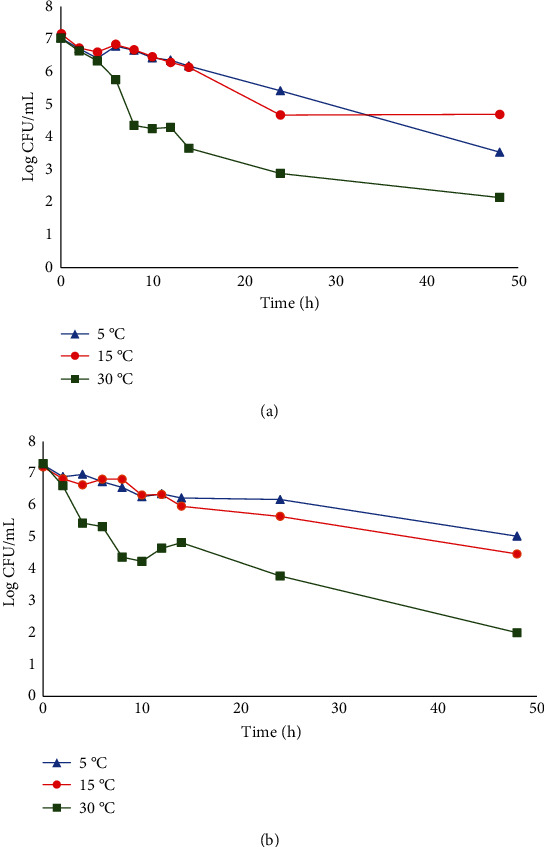
Growth rate of AAEC and NAEC in *Burkina* samples at different storage temperatures (5, 15, and 30°C): (a) AAEC (acid-adapted E. coli O157:H7) and (b) NAEC (nonadapted E. coli O157:H7).

There was a significant reduction in the counts of AAEC and NAEC in samples stored at 30°C compared to AAEC and NAEC in samples stored at 5°C and 15°C (Figure 1). AAEC and NAEC in *Burkina* stored at 30°C reduced by about 5 log CFU/mL within 48 h of storage at pH 4.03 and 4.02, respectively. At 5°C, there was a reduction of about 3 and 2 logs for AAEC and NAEC, respectively, compared to about 2 and 3 log reduction for AAEC and NAEC at 15°C. The lower reduction in counts observed for AAEC and NAEC in samples stored at 5 and 15°C could have been because the low temperatures protected the organisms much better than 30°C. At lower temperatures, vegetative cells are less metabolically active, so most stop growing to survive, making them less vulnerable to environmental stresses. Beales [21] reported that at cold temperatures, microorganisms experience metabolic imbalance and growth cuts due to the sensitivity of some metabolic processes. Due to the paused growth, cell concentration will not increase but will likely stay the same or slightly reduce with time. Although, the data favours storage at higher temperatures to reduce the growth of E. coli, for practical reasons including consumer acceptability, an initial pasteurisation step to reduce E. coli load would be advisable.

### 3.4. Survival Models

The highest *R*^2^ value of 0.97 was observed for AAEC in *Burkina* stored at 5°C for the primary models (Table 3). This value correlated with a low RMSE value of 0.187 and was statistically significant (*p* < 0.05). Though the *R*^2^ values for AAEC in *Burkina* at 15 and 30°C and NAEC in the sample stored at 30°C ranged from 0.744 to 0.79, the models obtained were statistically significant (*p* < 0.05). *R*^2^ is an index used to measure the output of a model, although other factors like *p* value and RMSE are used together to arrive at a good decision [22]. Stetz [23] provided a rule of thumb for an acceptable *R*^2^ as *R*^2^ > 0.75 for higher values of *R*^2^. All *R*^2^ values obtained were above 0.75 except the model developed for AAEC in *Burkina* stored at 30°C. Even though it was 0.744 which was less than 0.75, it was still close to 0.75 and can be considered high. Meanwhile, a low *R*^2^ does not mean a model is insignificant without assessing the corresponding *p* value [22]. For the model obtained for AAEC in *Burkina* at 30°C, the corresponding *p* value was 0.00133, making the model statistically significant. All *x* terms for primary models developed for the survival of AAEC and NAEC in *Burkina* stored at 5, 15, and 30°C (Table 3) were statistically significant. The only primary model that had two *x* terms was obtained for acid-adapted *E. coli* O157:H7 in *Burkina* stored at 30°C. Significance of the term further implies that as time increases, the counts of the organism decrease in product, with the decrease being faster at higher temperatures.

The predicted values obtained from the Baranyi model were comparable to the observed data (Tables 4 and 5). RMSE values calculated between the observed data and the predicted values by the Baranyi model ranged from 0.0007 to 0.8288. Chai and Draler [24] stated that RMSE is a good assessment tool for model evaluation.

The negative growth rate values obtained in Table 6 support the negative correlations observed between the bacterial count and storage time, which can be seen from the direction of the curves obtained in Figure 1. The highest rate value of -0.259 log CFU/mL/h was observed for AAEC in *Burkina* at 30°C. The rate obtained from the model is measured in log CFU/mL/h and could have a negative value indicative of death [25]. This implies the fastest rate at which counts of the bacteria are reduced in the product, with respect to storage time. Thus, as storage time increased, bacteria count reduced, further supporting the observation that the reduction in cell count was greater at higher temperatures.

## 4. Conclusion

From the study, it was observed that acid-adapted and nonadapted *Escherichia coli* O157:H7 in pasteurised *Burkina* stored at 5, 15, and 30°C survived after 48 h. However, they could not grow as indicated by decreasing counts with incubation time. No statistically significant difference existed between the AAEC and NAEC in *Burkina*, with AAEC and NAEC exhibiting acid tolerance.

Models explained 79% to 98% of the primary and secondary models developed from the RSME obtained. As time increases, the bacterium's survival decreases, decreasing faster at 30°C > 15°C > 5°C. The Baranyi model and the primary model developed explained the organism's survival, requiring the input parameter time (*x*) to predict its survival in *Burkina.*

Lower temperatures yielded higher counts of *E. coli* O157:H7 in the product, although concentrations decreased. From the models obtained, the concentration of *E. coli* O157:H7 in the product reduced as the storage time and temperature increased. Whereas storage at a higher temperature would not be advisable, an initial pasteurisation step to reduce microbial load is encouraged.

## Figures and Tables

**Table 1:** pH of acid-adapted and nonadapted 18 h broths.

	pH	
	Before inoculation	After inoculation
Acid-adapted (TSBG)	6.71 ± 0.01	4.75 ± 0.01
Nonadapted (TSB)	6.89 ± 0.00	6.14 ± 0.01

TSB: Tryptone Soya Broth; TSBG: Tryptone Soya Broth with 1% glucose.

**Table 2:** Effect of temperature and incubation time on the growth of acid-adapted and nonadapted *E. coli* O157:H7 in *Burkina*.

Time (h)	Log CFU/mL ± standard deviation					
	AAEC5	AAEC15	AAEC30	NAEC5	NAEC15	NAEC30
0	7.04 ± 0.03^a^	7.17 ± 0.04^a^	7.03 ± 0.12^a^	7.27 ± 0.19^a^	7.20 ± 0.11^a^	7.31 ± 0.01^a^
2	6.71 ± 0.02^ab^	6.73 ± 0.06^bc^	6.64 ± 0.12^a^	6.90 ± 0.11^ab^	6.83 ± 0.16^ab^	6.61 ± 0.23^b^
4	6.42 ± 0.57^ab^	6.61 ± 0.01^bc^	6.34 ± 0.06^ab^	6.97 ± 0.13^ab^	6.64 ± 0.29^abc^	5.44 ± 0.06^c^
6	6.79 ± 0.00^ab^	6.83 ± 0.10^b^	5.76 ± 0.40^b^	6.74 ± 0.09^bc^	6.82 ± 0.08^ab^	5.33 ± 0.03^c^
8	6.66 ± 0.02^ab^	6.68 ± 0.01^bc^	4.36 ± 0.12^c^	6.56 ± 0.01^bcd^	6.82 ± 0.23^ab^	4.37 ± 0.13^ef^
10	6.43 ± 0.00^ab^	6.47 ± 0.03^cd^	4.26 ± 0.12^c^	6.27 ± 0.12^cd^	6.32 ± 0.07^bcd^	4.24 ± 0.05^f^
12	6.36 ± 0.03^ab^	6.29 ± 0.03^de^	4.30 ± 0.14^c^	6.36 ± 0.03^cd^	6.34 ± 0.00^bcd^	4.65 ± 0.06^de^
14	6.18 ± 0.01^b^	6.14 ± 0.10^e^	3.66 ± 0.26^c^	6.23 ± 0.23^cd^	5.97 ± 0.35^cd^	4.83 ± 0.10^d^
24	5.42 ± 0.06^c^	4.68 ± 0.14^f^	2.89 ± 0.16^d^	6.18 ± 0.14^d^	5.65 ± 0.01^d^	3.78 ± 0.00^g^
48	3.54 ± 0.09^d^	4.70 ± 0.08^f^	2.15 ± 0.21^d^	5.03 ± 0.03^e^	4.47 ± 0.01^e^	2.00 ± 0.00^h^

Values are means and standard deviation from the duplicate analysis. Means that do not share a letter in the same column are statistically different across columns (*p* ≤ 0.05).

**Table 3:** Growth models for AAEC and NAEC in *Burkina*.

	Regression model	*R* ^2^ (adjusted)	RMSE	*p* value	CV error
AAEC5	*y* = 7.0623–0.0708*x*	0.97	0.187	2.28*e*‐07	0.0693
NAEC5	*y* = 6.9319–0.0549*x*	0.818	0.388	0.000322	0.5130
AAEC15	*y* = 7.0510–0.2823*x* + 0.0038*x*^2^	0.958	0.382	1.53*e*‐05	2.1166
NAEC15	*y* = 6.9845–0.0416*x*	0.918	0.187	1.28*e*‐05	0.0426
AAEC30	*y* = 7.0049–0.0546*x*	0.956	0.175	1.01*e*‐06	0.0479
NAEC30	*y* = 6.0418–0.0927*x*	0.79	0.763	0.00059	0.9248

*Y*: survival (log CFU/mL); AAEC: acid adapted *E. coli* O157:H7; NAEC: nonadapted *E. coli* O157:H7.

**Table 4:** Comparison of observed and predicted survival (log CFU/mL) of acid-adapted *E. coli* O157:H7 in *Burkina* at the selected storage temperatures (based on the Baranyi model).

Time (h)	5°C			15°C			30°C		
	Observed	Predicted	RMSE	Observed	Predicted	RMSE	Observed	Predicted	RMSE
0	7.045	7.062	0.0178	7.168	6.831	0.3367	7.033	7.096	0.0629
2	6.707	6.921	0.2137	6.726	6.827	0.1008	6.64	6.579	0.0616
4	6.423	6.779	0.356	6.608	6.815	0.2075	6.341	6.061	0.2794
6	6.785	6.637	0.1476	6.827	6.782	0.0449	5.759	5.544	0.215
8	6.658	6.496	0.1618	6.681	6.702	0.0206	4.363	5.028	0.6648
10	6.435	6.354	0.0804	6.47	6.545	0.075	4.259	4.514	0.2553
12	6.361	6.212	0.1486	6.286	6.314	0.0281	4.301	4.009	0.2921
14	6.185	6.071	0.1137	6.145	6.039	0.1057	3.661	3.527	0.1343
24	5.421	5.363	0.0585	4.679	4.752	0.0727	2.889	2.526	0.3628
48	3.540	3.663	0.1232	4.70	4.656	0.0437	2.151	2.513	0.3622

**Table 5:** Comparison of observed and predicted survival (log CFU/mL) of acid-non-adapted *E. coli* O157:H7 in *Burkina* at the selected storage temperatures (based on the Baranyi model).

Time (h)	5°C			15°C			30°C		
	Observed	Predicted	RMSE	Observed	Predicted	RMSE	Observed	Predicted	RMSE
0	7.266	6.985	0.2815	7.2	7.005	0.1951	7.308	6.598	0.7092
2	6.904	6.901	0.0027	6.834	6.896	0.0616	6.609	6.224	0.385
4	6.968	6.818	0.15	6.638	6.786	0.1484	5.442	5.866	0.4245
6	6.736	6.735	0.0007	6.816	6.677	0.1384	5.327	5.525	0.198
8	6.562	6.652	0.0895	6.816	6.568	0.2481	4.371	5.199	0.8288
10	6.272	6.568	0.2963	6.322	6.457	0.1371	4.242	4.891	0.649
12	6.363	6.485	0.1225	6.339	6.349	0.0104	4.646	4.598	0.0479
14	6.236	6.402	0.1657	5.97	6.240	0.2702	4.827	4.322	0.5049
24	6.178	5.986	0.1925	5.654	5.694	0.0404	3.778	3.188	0.5902
48	5.033	4.987	0.0466	4.47	4.383	0.0865	2	2.137	0.1369

**Table 6:** Parameter estimation (max growth rate) for acid-adapted and acid-non-adapted *E. coli* O157:H7 using the Baranyi model.

Sample	Rate (log CFU/mL/h)	*R* ^2^
AAEC5	-0.071	0.966
NAEC5	-0.042	0.908
AAEC15	-0.153	0.956
NAEC15	-0.055	0.951
AAEC30	-0.259	0.947
NAEC30	-0.093	0.763

AAEC = acid adapted *E. coli* O157:H7; NA = acid-non-adapted *E. coli* O157:H7.

## Data Availability

The data used to support the findings of this research are included in the article.
